# Perspectives about the baby friendly hospital/health initiative in Australia: an online survey

**DOI:** 10.1186/s13006-020-00266-z

**Published:** 2020-04-08

**Authors:** Anahita Esbati, Jane Taylor, Amanda Henderson, Margaret Barnes, Lauren Kearney

**Affiliations:** School of Nursing, Midwifery, and Paramedicine, University of the Sunshine Coast, Sippy Downs, Australia

**Keywords:** BFHI, Uptake, Implementation, Maternity facilities, Code of Marketing of Breast-milk Substitutes, Breastfeeding, Ten steps to successful breastfeeding

## Abstract

**Background:**

Evidence supports the health and economic benefits of breastfeeding, and the positive impact of the Baby Friendly Health Initiative (BFHI) on increasing breastfeeding rates and improving breastfeeding outcomes. The BFHI is a World Health Organization and United Nations International Children’s Emergency Fund initiative to promote, support and maintain breastfeeding. The BFHI was updated in 2018 and includes the *Ten Steps to Successful Breastfeeding* (the Ten Steps 2018) and the *International Code of Marketing of Breast-milk Substitutes* (the WHO Code). Despite policy and guideline support for breastfeeding and the BFHI, there are currently only 70 accredited hospitals, healthcare centres and regional clusters in Australia, accounting for 23% of maternity facilities. This research aimed to explore health professionals and other stakeholders’ perspectives on the uptake and implementation of the BFHI in Australia from an organisational change perspective.

**Methods:**

An online survey administered via relevant Australian-based professional associations was fully or partially completed by 332 participants who support mothers and newborns in their roles. Descriptive statistics and content analysis were used to analyse quantitative and qualitative data.

**Results:**

The majority of participants supported legislating the WHO Code, closely monitoring BFHI compliance, ensuring sufficient knowledge about breastfeeding and the BFHI among staff, improving care within maternity facilities, continuous support of mothers’ post-discharge, and improving social media support of breastfeeding. It was also perceived that an interdisciplinary team approach and multidisciplinary involvement were important requirements for successfully implementing the BFHI. There was no consensus among participants that Australian health policies support breastfeeding and the implementation of the BFHI.

**Conclusions:**

This study emphasised the significance of legislation around the Code, executive and leadership support and culture, and providing adequate resources concerning uptake and implementation of the BFHI. Considering that uptake of the BFHI has been limited and no formal government support has been provided to further develop the BFHI and support the Code in Australia, findings of this research may help with potential future actions to facilitate the BFHI uptake and Code implementation.

## Background

The health and economic benefits of breastfeeding for mothers, infants, and the broader community are well established [1, 2], however, breastfeeding rates remain suboptimal in Australia [3]. The most recent national data [2010] reported that only 15.6% of infants were exclusively breastfed to 5 months despite a high breastfeeding initiation rate (96%) [3]. Furthermore, 35% of infants were introduced to solid foods by 4 months of age and 92% by the recommended age of 6 months [3]. This low rate of exclusive breastfeeding, at less than the recommended 6 months of age, and considering that birth largely occurs in hospitals (97% of 309,000 births in 2015) [4] highlights the need for initiatives such as the Baby Friendly Hospital Initiative (BFHI) to support breastfeeding within maternity facilities [1, 2].

The BFHI is an initiative of the World Health Organization (WHO) and the United Nations International Children’s Emergency Fund (UNICEF) to protect, promote, and support breastfeeding [5] and has two parts: the *10 Steps to Successful Breastfeeding* (the 10 Steps) [5] and the *WHO International Code of Marketing of Breast-milk Substitutes* (the WHO Code) [6]. The aim of the 10 Steps, which was updated in 2018 (Table 1), is to ensure maternity services provide quality care for infants and essential support required to assist mothers to breastfeed [7]. The 2018 10 Steps is organised into two sections: *Critical management procedures* and *Clinical practices* [7]. New steps introduced in the update include Step 1a (Comply with the WHO Code and relevant resolutions), Step 1c (Establish ongoing monitoring and data-management systems), and Step 9 (Counsel mothers on the use of feeding bottles, teats, and pacifiers) [7]. Step 1a aims to ensure safe and adequate nutrition for infants by protecting breastfeeding and the proper use of breast milk substitutes [6, 7]. Step 9 aligns with literature that suggests a probable association between dummy use during sleep and a decrease in the risk of Sudden Infant Death Syndrome (SIDS) [8], even though it is recommended to delay dummy introduction until breastfeeding has been established [9]. Recent evidence also suggests that pacifier use started from birth or after lactation is established, did not influence exclusive or partial breastfeeding rates up to 4 months of age [10]. However, this finding was among mothers who were highly motivated to breastfeed and not all mothers [10].

**Table 1:** Ten Steps to Successful Breastfeeding updated in 2018 [6]

Critical management procedures	
1a.	Comply with the Code and relevant resolutions.
1b.	Have a written infant feeding policy that is routinely communicated to staff and parents.
1c.	Establish ongoing monitoring and data management systems.
2.	Ensure that staff have sufficient knowledge, competence and skills.
**Key clinical practices**	
3.	Discuss the importance and management of breastfeeding with pregnant women and their families.
4.	Facilitate immediate and uninterrupted skin-to-skin contact and support mothers to initiate breastfeeding after birth.
5.	Support mothers to initiate and maintain breastfeeding and manage common difficulties.
6.	Do not provide breastfed newborns any food or fluids other than breast milk, unless medically indicated.
7.	Enable mothers and their infants to remain together and to practise rooming-in 24 h a day.
8.	Support mothers to recognize and respond to their infant’s cues for feeding.
9.	Counsel mothers on the use and risks of feeding bottles, teats and pacifiers.
10.	Coordinate discharge so that parents and their infants have timely access to ongoing support and care.

Positive impacts of the BFHI on short, medium and longer-term breastfeeding outcomes are well established [11–13]. Examples of these impacts include increasing rates of breastfeeding initiation, duration, and exclusivity [12] and achieving exclusive breastfeeding intention [13]. An evidence review of 430 systematic reviews, qualitative, and quantitative studies brokered by the Sax Institute in 2018 also reported the BFHI to be the most effective intervention to improve rates of any breastfeeding and essential for breastfeeding exclusivity and duration [14].

Within Australia, the BFHI was launched in 1993 and its governance passed to the Australian College of Midwives by UNICEF in 1995 [15]. In 2009 the Australian Department of Health introduced The Australian National Breastfeeding Strategy (ANBS) (2010–2015) to support breastfeeding nationally [16]. Subsequent consultation in 2017 on the implementation of the ANBS recommended more widespread uptake of the BFHI to ensure the success of the ANBS [17]. The ANBS was updated in 2019 by the Australian Health Minister’s Advisory Council to provide an enabling environment for breastfeeding into the future [18].

Despite national support for breastfeeding and the BFHI [19], uptake within Australia is limited. There are only 70 accredited hospitals, healthcare centres, and regional clusters in Australia [20] accounting for approximately 23% of maternity facilities [21]. Understanding why uptake is limited is warranted, given the known benefits of breastfeeding and the effectiveness of the BFHI in improving breastfeeding rates. To implement the BFHI, hospital and health services may need to change relevant policies, structures, and practices to adhere to the conventions of the Initiative [22]. Understanding change components, implementation, and reorientation are required at both macro and micro levels, and organisational change models that systematically interrogate this change process are required [23].

The Burke and Litwin model (1992) (B-L model) underpinned by organisational change and performance was the framework used in this study to facilitate exploration of the elements of organisational change required to successfully implement the BFHI [23]. Based on this model, the external environment, relating to the macro level, is the most influential factor in organisational change [23]. Other components of the macro-level include leadership, organisational culture, and mission and strategy [23]. The micro-level encompasses management practices, structure, systems, work unit climate, motivation, task requirements and individual skills/abilities, and individual needs and values [23].

Recent Australian studies, including our own study, have identified barriers and facilitators related to the uptake and implementation of the BFHI at both macro and micro levels [21, 24, 25]. Examples of barriers to successful uptake and implementation in Australia are cultural and organisational obstacles [25], intangible government support, suboptimal capacity building to implement the BFHI [21, 25], and lack of established legal measures to implement the WHO Code [17]. Cultural and organisational obstacles may include old patterns, entrenched staff practices, staff rationalisation of noncompliance with the BFHI [25], and lack of essential resources [21, 25]. Facilitators to successfully implement the BFHI have been identified as leadership support [17, 21], improving BFHI and breastfeeding related knowledge, establishing better communication between stakeholders, facilitating the accreditation and reaccreditation process, and organisational cultural support of the BFHI according to findings of our research [24]. The findings of these studies align with and reinforce the 2018 version of ANBS, highlighting the significance of providing education and training, as well as restricting the advertising of infant formula [18]. The International Baby Food Action Network’s report of 2018 identified a lack of legal measures in Australia to implement the WHO Code [26]. This report suggested the establishment of a legal measure, along with essential enforcements, to implement the WHO Code and all its related resolutions [26].

Recent studies have explored the perspectives of Australian healthcare professionals or key stakeholders, who arguably influence directly or indirectly the macro and micro levels of change within organisations [17, 21]. However, gaps remain in our understanding of health professionals’ and stakeholders’ perspectives about the interrelation between barriers and facilitators influencing uptake and implementation of the BFHI from an organisational change perspective. There is also a paucity of larger quantitative studies to confirm or build on earlier qualitative exploratory studies. This research aimed to explore health professionals and other stakeholders’ perspectives on the uptake and implementation of the BFHI in Australia, specifically from an organisational change perspective.

## Methods

### Study design

An online survey was used to explore health professionals and stakeholders’ perspectives on the uptake and implementation of the BFHI in Australia from an organisational change perspective. At the time of the research, a reference to the 2009 version of the 10 Steps was used [5]. Therefore, participants had no knowledge of the 2018 guidance [7]. The development of the survey questionnaire was informed by the literature, a document analysis of current legislation and guidelines related to breastfeeding and the BFHI [19], and the results of interviews with health professionals about the BFHI conducted by authors [24]. The B-L model underpinned the survey structure to ensure both macro and micro levels of change implementation were addressed [23]. The survey included three sections:

1. Demographic characteristics: six questions including age, level of education, occupation, years of work experience, work experience involving caring for mothers and infants in the past month, and their principal state or territory of practice.
2. Uptake and implementation of the BFHI: 27 statements which assessed levels of agreement on a five-point scale. This section was informed by the previous study undertaken by this team, which identified six key themes, following a qualitative enquiry with semi-structured interviews [24]. This interview study explored health practitioners’ perspectives about uptake and implementation of the BFHI in Australia [24]. These six key themes included: 1) policy and guideline support and improvement for the BFHI, 2) leadership support to implement the BFHI, 3) improving breastfeeding and BFHI related knowledge, 4) accreditation and reaccreditation processes, 5) implementation complexity, and 6) communication between stakeholders regarding the BFHI.
3. One open-ended question as a general free comment was included at the end of the survey for participants to provide comments regarding the uptake and implementation of the BFHI in Australia.

The face validity of survey statements was examined by eight experts in the field and agreed amendments included. Experts were recruited through an initial email invitation. All ‘experts’ had achieved a Masters or PhD level qualification and > 5 years’ experience in education and/or research in the nursing and/or midwifery field. A total of eight participated in the face to face validity group meeting. A pilot test of the survey was then undertaken with a minimum target sample size of 30 to ensure the feasibility and quality of the survey [27]. To recruit potential participants to test the survey, health professionals who had participated in interviews previously [24] and those known within the network (health professionals with BFHI related experience) were sent an email invitation inviting them to participate and which included a link to the survey. Pilot participants were also invited to forward the invitation to those known to them (snowball sampling) [28]. Thirty-three responses (32 health and one non health professional) were received; 12 registered midwives (RMs), seven registered nurses (RNs), nine RN/RMs, 2 Child Health Nurses, one Paediatric nurse, one medical practitioner, one non-health professional Lactation Consultant (LC). Twelve health professionals were also LCs, three were researchers and one was involved in BFHI accreditation besides their professional roles. Responses informed minor changes to the survey, for example, amendments to the wording of questions to enhance clarity.

### Population and sample

Convenience and snowball sampling were used to recruit participants from relevant health professional (such as the Australian College of Midwives (ACM)) and stakeholder (such as the Australian Breastfeeding Association (ABA) and Homebirth Australia) organisations [28]. Considering the BFHI implementation involves a range of health professionals within facilities to implement Steps 1–9, as well as stakeholders outside facilities to implement Step 10, this study targeted a wide range of health professionals and stakeholders to capture their perspectives. Health professionals included RNs, RMs, RNs/RMs, Maternal and Child Health Nurses, LCs, doctors, nutritionists, dietitians, community health managers, physiotherapists, complementary therapists, and a lab technician. Other respondents identified as breastfeeding advocates such as breastfeeding counsellors, peers who support mothers and newborns, and other related professionals (e.g. herbalist). The initial survey target sample size was at least 100 respondents to ensure that the sample size was appropriate for the planned analysis [29].

### Recruitment and data collection

Relevant Australian-based stakeholders and health professional networks mentioned above were invited to participate in the research via email and/or phone using contact information publicly available on their websites. Nine organisations agreed to share the invitation with their members via website pages, e-newsletters, and/or Facebook pages. A link to the survey was included in the invitation which was administered between June and November 2017 using SurveyMonkey Software. Two strategies were used to conduct an audit trail; 1) checking the Internet Protocol (IP) addresses, and 2) the Survey monkey platform was set to block participants from completing the survey a second time to prevent repetition.

### Data analysis

Descriptive frequencies were used to analyse agreement scale data using SPSS Statistics (version 24) [30]. Content analysis of responses to the open-ended question (general free comment) was completed [31] using NVivo software (version 10) as a data and coding manager program [32]. There were 73 free response comments totalling 5820 words. Responses to open-ended questions were read by the researcher (AE). Meaningful data segments were then identified, from which a group of related codes was drawn to inform the development of key concepts. Examples of participant statements related to these codes and key concepts were checked and validated by the research team. Key concepts were then mapped to six main key areas underpinned by a previous study that interviewed 12 health practitioners - conducted by authors of this study [24].

### Ethical considerations

The study was approved by the University of the Sunshine Coast Human Research Ethics Committee (S/15/806). Potential participants were provided information regarding the survey which was attached to the first page. Participation in the survey was voluntary and participants’ responses anonymous. Consent was implied upon completion of the survey.

## Results

A total of 343 survey responses were received, of which 165 were fully and 178 partially completed. Lack of exposure to experience (such as working within a Baby-Friendly hospital) may have contributed to moderate rates of non-completion of the survey. Eleven participants’ responses (including mothers and students) were excluded as consumer perspectives were beyond the scope of this study. A total of 332 returned survey responses were included in the data analysis.

Results are presented in two sections: demographic characteristics (Table 2) and participants perspectives on the uptake and implementation of the BFHI in Australia (Tables 3 and 4).

**Table 2:** Participant characteristics

Variable	Frequency	Percentage (%)
Age (*n* = 169)		
20–40 years	47	27.8
40–60 years	105	62.1
Over 60 years	17	10.1
Level of Education (*n* = 169)		
High school	2	1.2
Some college-tertiary education	1	0.6
Diploma	10	5.9
Undergraduate degree	37	21.9
Postgraduate degree	119	70.4
Occupation (*n* = 163)		
Nurses and/or midwives (*n* = 91)		
RN-RM	32	19.6
RM	28	17.2
RN	9	5.5
CFH Nurse	9	5.5
Clinical Midwife Consultant (CMC)	4	2.5
NUM	3	1.8
Paed Nurse	3	1.8
CFH Nurse- Paed Nurse	2	1.2
Enrolled nurse	1	0.6
LCs & peer supporters (*n* = 38)		
Lactation consultants	31	19.0
Peer supporters	7	4.3
Research, education, accreditation (*n* = 25)		
Educators	10	6.1
Researchers	9	5.5
BFHI accreditors	6	3.7
Other health professions (*n* = 6)		
Senior policy officer	1	0.6
Medical practitioner	1	0.6
Aboriginal health officer	1	0.6
Laboratory technician (breastfeeding counsellor)*	1	0.6
Psychologist (doula)	1	0.6
Chiropractor	1	0.6
Other professions (*n* = 3)		
Herbalist (home birth)	1	0.6
Community worker	1	0.6
Manager (not specified)	1	0.6
Years of work experience (*n* = 156)		
None	2	1.3
Less than 1 year	5	3.2
1–5 years	17	10.9
6–10 years	28	17.9
11–20 years	43	27.6
Over 20 years	61	39.1
Work experience in the past month^#^ (*n* = 162)		
0–8 h	26	16
1–5 days	21	13
6–10 days	21	13
11–20 days	76	46.9
21–30 days	18	11.1
State or Territory of practice (*n* = 169)		
Australian Capital Territory	6	3.6
Northern Territory	1	0.3
New South Wales	67	39.6
Queensland	45	26.5
South Australia	10	5.9
Tasmania	13	7.7
Victoria	16	9.5
Western Australia	11	6.5

*Breastfeeding counsellors are trained volunteers who provide breastfeeding support to mothers via phone, email, local support group meetings, and/or local support group. To provide such support, they must have breastfed at least one baby, hold a current Certificate IV in Breastfeeding Education (Counselling) or equivalent, and complete ongoing training [ref: https://www.breastfeeding.asn.au/roles/breastfeeding-counsellors]

^#^Hours and/or days of work experience relate to care of mothers and infants in the past month

**Table 3:** Survey results

Statements	Strongly disagree ***n*** (%)	Disagree ***n*** (%)	Neutral ***n*** (%)	Agree ***n*** (%)	Strongly agree ***n*** (%)
1. Policy and guideline support and improvement of breastfeeding and the BFHI					
Australian health policies support breastfeeding as a public health issue. (*n* = 234)	10 (4.3)	63 (26.9)	60 (25.6)	79 (33.8)	22 (9.4)
Australian health policies support implementation of the BFHI. (*n* = 235)	13 (5.5)	55 (23.4)	80 (34.0)	71 (30.2)	16 (6.8)
Advertising for toddler formula should be banned in Australia. (*n* = 191)	6 (3.1)	16 (8.4)	7 (3.7)	28 (14.7)	134 (70.2)
The *Code of Marketing of Breast-milk Substitutes* should be legislated in Australia to improve compliance. (*n* = 192)	2 (1.0)	4 (2.1)	11 (5.7)	45 (23.4)	130 (67.7)
Research funded by formula companies should be supervised by a governing body to ensure compliance with the *Code of Marketing of Breast-milk Substitutes*. (*n* = 192)	0 (0)	1 (.5)	12 (6.3)	44 (22.9)	135 (70.3)
Most maternity facilities in Australia adhere to the *Code of Marketing of Breast-milk Substitutes*. (*n* = 192)	17 (8.9)	40 (20.8)	84 (43.8)	46 (24.0)	5 (2.6)
Current breastfeeding guidelines provide adequate details about when it is medically essential to provide formula for infants. (*n* = 192)	22 (11.5)	61 (31.8)	42 (21.9)	57 (29.7)	10 (5.2)
The BFHI guidelines address cultural differences in feeding practices. (*n* = 195)	5 (2.6)	62 (31.8)	87 (44.6)	38 (19.5)	3 (1.5)
2. Leadership support to implement the BFHI					
Organisational leadership influences implementation of the BFHI. (*n* = 237)	4 (1.7)	1 (.4)	19 (8.0)	69 (29.1)	144 (60.8)
Mothers receive adequate support for breastfeeding postnatally (*n* = 188)	54 (28.7)	79 (42.0)	28 (14.9)	21 (11.2)	6 (3.2)
3. Breastfeeding and BFHI related knowledge					
Up-to-date educational resources are freely available for staff to support implementation of the BFHI. (*n* = 236)	6 (2.5)	47 (19.9)	58 (25.0)	74 (31.4)	50 (21.2)
Breastfeeding-related education should be compulsory for staff involved in caring for mothers and babies. (*n* = 236)	2 (.8)	2 (.8)	2 (.8)	30 (12.7)	200 (84.7)
Mothers are provided with information by healthcare staff about how and where to access appropriate breastfeeding resources. (*n* = 235)	7 (3.0)	37 (15.7)	45 (19.1)	101 (43.0)	45 (19.1)
Healthcare professionals are aware of the benefits of implementing the Ten Steps. (*n* = 191)	18 (9.4)	58 (30.4)	45 (23.6)	59 (30.9)	11 (5.8)
4. Accreditation and reaccreditation					
BFHI accreditation is not essential if appropriate practices (implementing the Ten Steps) exist. (*n* = 194)	26 (13.4)	66 (34.0)	36 (18.6)	52 (26.8)	14 (7.2)
The Ten Steps are consistent with current evidence-based practice on breastfeeding. (*n* = 195)	1 (.5)	6 (3.1)	18 (9.2)	115 (59.0)	55 (28.2)
In settings where the initiation rate of breastfeeding is high, implementation of the BFHI has less impact. (*n* = 194)	23 (11.9)	81 (41.8)	52 (26.8)	33 (17.0)	5 (2.6)
Formal organisational structures are required within maternity settings to monitor and evaluate the implementation of the BFHI. (*n* = 236)	1 (.4)	6 (2.5)	11 (4.7)	101 (42.8)	117 (49.6)
Changes to current models of maternity care may be more influential in improving breastfeeding outcomes than BFHI accreditation. (*n* = 195)	4 (2.1)	28 (14.4)	41 (21.0)	77 (39.5)	45 (23.1)
The financial cost of the BFHI accreditation for maternity settings is worth the health outcomes for mothers and infants. (*n* = 196)	4 (2.0)	23 (11.7)	50 (25.5)	59 (30.1)	60 (30.6)
Feedback from a range of key stakeholders (e.g. mothers and/or interdisciplinary staff) is essential for successful implementation of the BFHI. (*n* = 191)	1 (.5)	0 (0)	6 (3.1)	89 (46.6)	95 (49.7)
Continuous monitoring of BFHI accredited maternity facilities is essential to ensure ongoing compliance by these health facilities. (*n* = 191)	0 (0)	3 (1.6)	10 (5.2)	95 (49.7)	83 (43.5)
5. Implementation complexity					
The choice to breastfeed is influenced by a mother’s values about breastfeeding. (*n* = 188)	2 (1.1)	12 (6.4)	6 (3.2)	102 (54.3)	66 (35.1)
Support for breastfeeding by social media influences mothers’ breastfeeding decisions. (*n* = 188)	0 (0)	7 (3.7)	20 (10.6)	102 (54.3)	59 (31.4)
Breastfeeding in public is supported in the Australian context. (*n* = 188)	13 (6.9)	65 (34.6)	52 (27.7)	53 (28.2)	5 (2.7)
Societal acceptance of breastfeeding influences mothers breastfeeding decisions. (*n* = 188)	0 (0)	4 (2.1)	11 (5.9)	88 (46.8)	85 (45.2)
Communication among stakeholders					
An interdisciplinary team approach is required for successful implementation of the BFHI. (*n* = 236)	2 (.8)	4 (1.7)	8 (3.4)	73 (30.9)	149 (63.1)

**Table 4:** Content analysis from ‘free text’ response in survey

Statement	Key concept from participant response	Example verbatim text
Policy and guideline support and improvement of breastfeeding and the BFHI	1. Lack of national support of the WHO Code	1.1. ‘*National support is lacking strength for this especially in relation to MAIF* [Manufacturers and Importers Agreement 1992].’ (Participant 48) 1.2 *‘I believe that we should embrace all recommendations from the WHO when it comes to BFI/BFHI including stopping the promotion and advertising of any formula (newborn or toddler milks).’* (Participant 263)
	2. A need for policy support for the BFHI	2.1 ‘*I believe that at a policy level BFHI needs to be a mandatory accreditation.*’ (Participant 314)
	3. A need for policy and/or guideline improvement.	3.1 ‘*The increased documentation of compliance is time consuming, resented by staff, and makes mothers feel like they are being excessively scrutinised … the BFHI compliance regulations are becoming counterproductive to breastfeeding promotion*.’ (Participant 85)
Leadership support to implement the BFHI	1. A need for government support	1.1 ‘*The uptake of BFHI requires high level governance and funding at a government level in every aspect of health to effect any change*.’ (Participant 106)
	2. A need for support by senior executive health staff	2.1′*Senior executive health staff do not prioritise implementation of BFHI sufficiently in my area*.’ (Participant 38)
	3. Supporting mothers	3.1 *‘Peer support and referral to organisations such as the Australian Breastfeeding Association should be encouraged and nurtured.’* (Participant 43)
Breastfeeding and BFHI related knowledge	1. Lack of essential knowledge and skills amongst staff	1.1 ‘*Our health professionals’ breastfeeding advice is inconsistent and out-dated at best.*’ (Participant 26)
	2. A need for updated training and required qualifications	2.1 ‘*I recall when the initiative was first implemented in our health service. All staff were trained. Since then I have not seen any updates or requirement to re-accredit, which would lead to new staff not being aware of the policy.*’ (Participant 87)
	3. A need for easy access to education	3.1 ‘*Easy access to staff education and staff support is a key component to BFHI*.’ (Participant 47)
	4. Providing education for mothers and families	4.1 ‘*More recognition of long-term health benefits of breastfeeding to society by government*. *.*. *and education services with resources to enable education and support for individuals and families to be empowered to feed their babies*.’ (Participant 121)
	5. A need for knowledge improvement about breastfeeding in the community	5.1 ‘*There also needs to be more community awareness around breastfeeding.*’ (Participant 135)
Accreditation and reaccreditation	1. More BFHI accredited facilities are required	1.1 & 2.1 ‘*More hospitals should be BFHI accredited and those who already are should be more closely monitored*.’ (Participant 1)
	2. A need for monitoring	As Above
	3. Cost of accreditation	3–1 ‘*It’s very expensive for hospitals to achieve accreditation*.’ (Participant 56)
	4. BFHI was not considered essential and/or beneficial	4.1 ‘*I do find BFHI accreditation restrictive to midwifery practice and can serve to alienate some mothers by not supporting their informed decision making*.’ (Participant 92)
Implementation complexity	1. Staff attitudes towards breastfeeding and the BFHI	1.1 ‘*I think the management of the hospitals and maternity units need to see breastfeeding as valuable to go for BFHI accreditation*.’ (Participant 324)
	2. Community attitudes and support towards breastfeeding	2.1 ‘*I was still subjected to public criticism when breastfeeding my infants in public*. *.*. *so I don’t think as a society breastfeeding is as well accepted as it should be*.’ (Participant 88)
	3. Media support of breastfeeding	3.3 ‘*Media needs to support breastfeeding and portray it as the normal*.’ (Participant 136)
Communication among stakeholders	1. Importance of a multidisciplinary approach to the BFHI	1.1 ‘*BFHI needs to be multidisciplinary and led by national government*. *.*. (Participant 60)
	2. Importance of a continuous relationship between mothers and healthcare staff	2.1 ‘*Maternal child health nurses have an ongoing relationship with the mother and baby and are more likely to influence continued breastfeeding than* [the] *BFHI*.’ (Participant 163)
	3. Importance of communication skills and/or interaction between healthcare staff and mothers	3.1 ‘*I am often disappointed by what can be seen as bullying type practices from midwives, and almost inflexible militant type approach to upholding BFHI practices. This often is a bone of contention and actually put women off breastfeeding as they feel they can’t live up to the expectations of the inflexible breastfeeding advocate*.’ (Participant 105)
A need for improvement in care^a^	1. A need for improving care provided for mothers’ post discharge	1.1 ‘*To me this* means *BFHI is working, but more programs are needed to support women after they go home from hospital*.’ (Participant 166) 1.2 *‘Postnatal care is the least well-regarded part of birthing continuum and least well resourced.’* (Participant 108) 1.3 *‘BFHI is great but so many mothers are discharged on day two, and support services following are not adequate.’* (Participant 319) 1.4 *‘Community BFHI needs more emphasis.’* (Participant 96) 1.5 ‘*Also more continuity of care models would improve breastfeeding rates.*’ (Participant 141)
	2. A need for improved care provided by hospitals	2.1 *‘Hospitals should refer more readily to the community-based family care.’* (Participant 27) 2.2 ‘*More hospitals should have breast milk storage banks. To provide babies in NICU and SCN donor breast milk as an alternative to formula.*’ (Participant 141) 2.3 *‘Women need far more support to initiate breast feeding in a hospital birth context.’* (Participant 173) 2.4 *‘Early discharge from hospital lessens breastfeeding support*. *.*. *More IBCLCs are required in hospitals and CFHN clinics for early breastfeeding support.’* (Participant 334)

^a^This statement was constructed in response to the ‘free text’ question at the end of the survey

### Demographic characteristics

The majority of participants were between 40 and 60 years old (*n* = 105; 62%) and had a postgraduate degree (*n* = 119; 70.4%). Just over half (*n* = 91; 55.7% were RN, RM or RN/RM) and 39.1% (*n* = 61) had over 20 years of work experience. Almost 46.9% (*n* = 76) had between 11 to 20 days of work experience caring for mothers and infants in the past month, and over one third practised in the state of NSW (*n* = 67; 39.6%) (Table 2).

### Participants perspectives on the uptake and implementation of the BFHI in Australia

Survey results are presented for each of the six key areas examined in the survey in Table 3 (Likert scale responses) and Table 4 (free-text responses). There was no consensus that Australian health policies support breastfeeding or implementation of the BFHI, or that implementation was a priority. The greatest proportion of stakeholders (*n* = 84; 43.8%) were neutral about maternity facility adherence to the Code. Findings also identified that some hospitals breach the Code due to a lack of national support for its implementation. Participants suggested the Code be legislated to improve compliance and research funded by formula companies to be supervised by a governing body. It was also perceived that close monitoring, ensuring sufficient knowledge among staff, interdisciplinary and multidisciplinary involvement, improving care within maternity facilities, continuous support of mothers’ post-discharge, and improving social media support of breastfeeding were influential on the uptake and implementation of the BFHI.

The majority of participants (*n* = 213; 89.7%) agreed or strongly agreed that organisational leadership influences the uptake and implementation of the BFHI and that a formal organisational structure is required for successful implementation (*n* = 218; 92.4%) (Table 3). Most participants (*n* = 133; 70.7%) disagreed or strongly disagreed that mothers receive adequate support for breastfeeding postnatally and suggested a need for Government and senior executive health staff to support the implementation of the BFHI (Table 3).

There was no consensus among participants about the level of staff awareness of the benefits of implementing the 10 Steps, even though half (*n* = 124; 52.5%) agreed or strongly agreed that up-to-date educational resources are freely available for staff to support implementation. Participants commented that practice is often not aligned to the BFHI standards. Issues identified include misleading advice on breastfeeding by health staff (such as the use of supplementation to increase weight gain in the case of weight loss or jaundice) and inconsistency in the interpretation of the standards by healthcare staff.

Participants identified that the financial cost of the BFHI accreditation for maternity settings is worth the long-term health outcomes for mothers and infants. Participants also commented that accreditation is the gold standard, implying more BFHI accredited facilities are required.

Identified barriers included a lack of funding for facilities to become accredited, the cost of education and implementation fees. Participants identified that it was important to support the continuous monitoring of BFHI accredited maternity facilities and collect feedback from a range of key participants to ensure continuous compliance. The majority (*n* = 122; 62.6%) of participants agreed or strongly agreed that changes to current models of maternity care may be more influential on improving breastfeeding outcomes than the BFHI accreditation. Participants’ comments also identified that the BFHI is ‘restrictive to midwifery practice’.

The majority of participants agreed or strongly agreed that the decision to breastfeed is influenced by a mother’s values about breastfeeding (*n* = 168; 89.3%), societal acceptance of breastfeeding (*n* = 173; 92%), and support for breastfeeding within social media (*n* = 161; 85.6%). There was no consensus as to whether breastfeeding in public is supported in Australia.

Most participants (*n* = 222; 94.1%) agreed or strongly agreed that an interdisciplinary team approach is required for successful implementation of the BFHI and that post-discharge care should be enhanced to improve the duration of feeding.

## Discussion

This study has identified health professionals’ and stakeholders’ perspectives within Australia on the uptake and implementation of the BFHI from an organisational change perspective. This is the first Australian study in which a broad range of participants from all states and territories participated in an online survey, and its findings were explored through the B-L model [23]. The findings identify facilitators and barriers at the macro and micro levels of this model (Table 5).

**Table 5:** Components of Burke-Litwin model (1992), related findings, and recommendations to facilitate uptake and implementation of the BFHI

Macro level		
Components related to the BL model	Findings related to each component	Recommendation examples
1. External environment	1.1 Influence of infant formula industry via their formula promotion advertising 1.2 Influence of societal attitudes 1.3 Influence of social media	● Minimising the impact of formula promotion on mothers, families, and communities ● Ensuring that formula industry act responsibly within social media context ● Improving public support of breastfeeding by improving societal knowledge and acceptance of breastfeeding ● Improving the breastfeeding messages on social media
2. Leadership support	2.1 Government support and intervention 2.2 Policy support and implementation to facilitate uptake and implementation of the BFHI 2.3 Organisational leadership to facilitate uptake and implementation of the BFHI	● Government intervention to promote the BFHI ● Establishment of supportive policies to facilitate uptake and implementation of the BFHI ● Evaluating and improving current policies and guidelines ● Strengthening the WHO Code implementation by legislation ● Develop and engage credible leadership to implement change ● Providing adequate and essential resources
3. Mission and strategies	3.1 Setting strategies to achieve the BFHI accreditation 3.2 Setting strategies to maintain reaccreditation 3.3 Establishment of plans to check feedbacks to ensure maintenance of Ten Steps	● Increasing number of accredited facilities ● Prioritising facilities with lower than optimum breastfeeding rates (e.g. initiation rate) for potential BFHI accreditation ● Prioritising facilities where best standard (Ten Steps) are not well established and/or practiced for potential BFHI accreditation ● Checking feedback from health professionals, stakeholders, and consumers
4. Organisation culture	4.1 Organisational culture support of the BFHI uptake and implementation	● Organisational cultural change -changing attitudes and practices-might be essential to implement the BFHI ● Staff attitudes towards breastfeeding must align with the BFHI standards
**Micro level**		
5. Management practices	5.1 Supportive management to implement the BFHI	● Develop and engage reliable and supportive management ● Managers must support staff to implement the BFHI by allocating required resources (e.g. free available educational materials) within facilities
6. Systems	6.1 Promotion of multidisciplinary team involvement 6.2 Establishment of an interdisciplinary team approach 6.3 Improvement in referral systems	● Establishing strategies to harmonize involvement among professional groups towards BFHI ● Improving inter professional collaboration ● Ensuring the referral of mothers to supportive breastfeeding groups post discharge to ensure continuous care
7. Structure	7.1 Continuous monitoring of the BFHI 7.2 Implementing structural change within related facilities to improve care 7.3 Implementing structural change in models of care to improve care	● Establishment of formal organisational structures to evaluate and/or monitor implementation of the BFHI ● Establishment and promotion of breast-milk banks within maternity facilities ● Promotion of continuous midwifery models of care
8. Work unit climate	8.1 Improving communication and/or interaction skills between healthcare staff and mothers	● Providing a continuous relationship between mothers and staff during the care period ● Improving communication skills between healthcare staff and mothers ● Improving interaction skills between healthcare staff and mothers
9. Motivation	9.1 Motivating organisations and/or individuals to facilitate uptake and implementation of the BFHI	● Reinforcing the public health impacts of the BFHI to motivate individuals and organisations ● Using motivating agents to ensure forward movement with the BFHI implementation
10. Task requirements and individual skills/abilities	10.1 Improving breastfeeding and BFHI related knowledge amongst staff, mothers, family members, and community	● Educating staff about the benefits of the Ten Steps ● Establishment of compulsory breastfeeding training programs for staff ● Providing up to dated and easily accessible training resources for staff
11. Individual needs and values	11.1 Addressing mothers’ needs and values 11.2 Addressing staff’s needs and values	● Addressing cultural differences in BFHI guidelines ● Providing culturally appropriate compassionate care for mothers ● Identifying staff needs to provide resources accordingly
12. Individual and organisational performance	12.1 Successful uptake and/or implementation of the BFHI	● Achieving successful uptake of the BFHI ● Achieving successful implementation of the BFHI

A key finding of this study was the perceived impact of the external environment (relevant to macro-level) on the BFHI adoption through formula advertising, social norms, and social media. The 2016 Lancet review reported that France and the US are expected to have a negative growth rate in the sale of formula products (follow-on and toddler milks) due to the legislation, public awareness campaigns, and actions by society [1]. However, in other high-income countries, an increase in sales of these formula products is expected due to the advertising activities [1] while Australia lacks legislation to empower the Code implementation [17]. Our study found that the capacity to adopt BFHI practices is negatively affected by the lack of legal measures to reinforce the Code and monitor formula industry activities. The negative impact of formula advertisement, particularly the promotion of toddler formula on mothers’ breastfeeding decisions, was identified specifically in our study which also aligns with another Australian study [33]. Another recent Australian study also reported that government efforts to prohibit health and nutrition claims within the advertising of formula have been ineffective [34].

Participants identified that support from wider society is required to see breastfeeding more as the norm rather than an offensive public act, which aligns with a 2019 narrative review that recommended community support of breastfeeding [35]. However, breastfeeding in public appears an issue in Australia [36] and the US [37]. A recent Australian study that explored 15 family conversations regarding breastfeeding identified several concerns [36]. These concerns included that women are required to be covered-up, find an appropriate place to avoid discomforting others, and guard against the judgement, to be able to breastfeed in public [36]. This is similar to studies conducted in the US that found existing negative community practices were a barrier to the BFHI implementation [37]; with subsequent need for a change in community views about breastfeeding recommended [38].

While a recent study identified that changes are needed to current breastfeeding messages to normalise breastfeeding and emphasise wider values rather than only health benefits [39], participants’ responses here have echoed international findings emphasising the “normalisation” of breastfeeding. It was perceived by the majority of participants that support for breastfeeding by social media influences mothers’ breastfeeding decisions while the Sax review reported limited evidence that cultural norms can be influenced through mass media and social marketing campaigns [14]. As such, future research to assess the potential impact of social media on the BFHI adoption and implementation would be helpful.

Our study identified the role of leadership support as a vital component concerning policy establishment and building a ‘BFHI culture’ at macro and micro levels. The findings identified no consensus among participants that Australian policies support breastfeeding and the BFHI. Another Australian study also identified national breastfeeding statements as ‘soft’ policy due to the lack of tangible incentives or measurable outcomes [21, 40]. In regards to establishing a BFHI culture, recent literature has also identified the impact of leadership support at the government [40] and organisational levels on the uptake and implementation of the BFHI [37, 41] via providing required resources as enablers [42] while ‘intangible government support’ and ‘suboptimal capacity building’ and resource issues flag barriers [21, 25, 43]. Other barriers to the successful establishment of the BFHI culture are difficulties associated with changing existing practices of hospitals [37] or resistance to new policies [43] and therefore, a change in practice and attitude by staff is required [38].

While resource issues for accreditation have been identified as a barrier for capacity building to achieve accreditation [21, 25], findings of our study identified no consensus among participants concerning the necessity for accreditation. However, the 2016 Lancet review recommended the establishment of legislation that all maternity services adhere to the BFHI [1]. While it was perceived that essential resources are available for the education of staff, there was no consensus among participants concerning staffs’ level of awareness of the benefits of the 10 Steps. A gap was also perceived between staff practices and BFHI standards, emphasising the importance of monitoring to ensure staffs’ use of BFHI related educational resources to prevent outdated and inconsistent practices.

In regards to micro-level components, participants identified that engagement of supportive management, structural changes, collaboration via interdisciplinary and multidisciplinary involvement, establishing quality communication, and information sharing would help move the BFHI forward. Capacity building for management practices within Australian facilities may encounter difficulties due to the lack of resources [21, 25]. However, government financial investment to support breastfeeding has been emphasised in the Sax and 2016 Lancet reviews [1, 14]. A recent study identified the provision of providing authority, motivation, and resources for managers, as well as more collegiality between the different units within a hospital, are requirements to support the BFHI implementation [44].

Our findings also suggested improving structures alongside the BFHI accreditation, such as the integration of Human Milk Banks (HMBs) within hospitals or upscaling midwifery continuity of care would be beneficial. The establishment of HMBs has been found to strengthen the BFHI effectiveness [45]. Similarly, continuity of midwifery care is known to be associated with improved maternal and neonatal outcomes [46, 47] including increased breastfeeding initiation [48]. Models of midwifery care facilitating relationship building between mother and midwife as well as mother and infant [48], which are practical, consistent, and family-centred, are encouraged as such models have a positive impact on breastfeeding outcomes [49].

The importance of establishing quality communication and interaction skills and its potential impact on the dissemination of information at organisational and individual levels was also highlighted. This study found that disseminating the message of the public health impacts of the BFHI to organisations (leadership) and individuals (staff and managers) would motivate uptake and implementation of the BFHI. To successfully share such messages with mothers, there is a need for establishing quality communication between healthcare staff and mothers, which is based on trust [49, 50]. These studies identified that women need to feel that staff spend sufficient time with them so they feel listened to, while lack of time by staff has been identified as a major barrier [49, 50]. An Australian study also identified that communication is vital in supporting a mother to feel confident in her knowledge about her and her infant’s needs [48]. In this study, considering women to be knowledgeable about these needs was perceived as an enabler [48]. Our study also emphasised the involvement of the mothers’ family members and wider society in information sharing interventions as they play an important role in mothers breastfeeding decisions. This finding is also supported by a recent study which found that positive influential factors on the BFHI implementation included the integration of education and interpersonal support, with involvement of women’s partners and family, in maternity care interventions [25].

Consideration of a new mother’s values and needs (micro-level) was seen as very important, aligning with recent literature suggesting culturally tailored services are significant [51]. A mixed-method analysis of five qualitative studies found that BFHI may not address women’s needs and cause negative emotional experiences [49]. These negative experiences were due to poor communication and attitudes by staff which included the use of judgemental language, criticism of feeding attempts, unrealistic expectations, and using discourse which may cause feelings of guilt in breastfeeding women. The findings of these studies reinforce the importance of establishing communication based on trust, maternal values, and family involvement to ensure a supportive environment is provided for breastfeeding mothers within accredited facilities.

Overall, our findings identified that interventions at macro and micro levels are required in Australia to establish capacity for future potential improvement in uptake and implementation of the BFHI. Achieving this goal is not only highly resource-dependent requiring government support, but also needs attention paid to influential factors in the external environment as this is also identified in the Lancet 2016 report [1]. The findings of our study also align with the critical management procedures and clinical practice components of the updated 10 Steps 2018 even though at the time of this study participants’ knowledge was based on the earlier version of 10 Steps (2009) [5]. While the 2018 10 Steps address the commentary around the WHO Code, this potentially needs to be supported by legislation in Australia to strengthen implementation and ensure compliance, related to Step 1a of 10 Steps 2018 [7].

These influential factors relevant to the 12 components of the B-L model are interrelated [23], reflecting the multifaceted nature of the BFHI uptake and implementation. Implementation of each component could potentially influence the application of other components, impacting on individual and organisational performance (final product) [23], which is the uptake and implementation of the BFHI.

The integration of this model with findings of this study could potentially provide an effective strategy for managing organisational change within facilities intending to become accredited. This might be achieved by initially identifying and analysing barriers related to the B-L component/s influencing the change within organisations [23]. This could then be followed by the establishment of essential action plans to move forward with the BFHI adoption and implementation.

### Strengths and limitations

Strengths of this study include the application of a framework [23] to integrate the quantitative findings to the context of BFHI uptake and implementation, and the inclusion of a broad participant group with representatives from a diverse, yet significant group of health professionals and stakeholders. The survey was informed through a methodological sound process and directly informed by qualitative interviews conducted with health practitioners with expertise in BFHI uptake and implementation [24] .

However, a convenience sample limits the generalisability of the findings from this study to the broader population and the survey did not include a measure of participant exposure to the BFHI, increasing the possibility of bias in results. Some incomplete responses were present in the cohort, limiting the reliability and validity of results [52]. Another limitation of this study was the use of free comment lacking some of the key strengths of qualitative research even though it may increase response rates and may identify issues which complement responses to closed statements [53].

## Conclusions

Despite the known health and economic benefits of breastfeeding, and the effectiveness of interventions such as the BFHI to improve breastfeeding rates, uptake in Australia remains sub-optimal. Integration of participants’ perspectives and theoretically-based organisational change theory (in this instance the B-L model) was undertaken for this study, and for the first time in the Australian context. A range of health professionals and stakeholders agreed macro-level factors, such as legislation around the Code, executive and leadership support and culture, as well as adequate resourcing are strongly influence the uptake and implementation of the BFHI. There was no consensus among participants that Australian health policies support breastfeeding and the implementation of the BFHI, despite the BFHI being considered a key mechanism to promote and support breastfeeding. Considering that uptake of the BFHI has been limited and no formal government support has been provided to further develop the BFHI and support the WHO Code in Australia, despite the recommendations of the NBSF, findings of this research may help with potential future actions to facilitate the BFHI uptake and Code implementation.

## Data Availability

The datasets generated and/or analysed during the current study are not publicly available to protect any potential identification of individuals and/or maternity facilitates represented in the data; but are available from the corresponding author on reasonable request.

## Abbreviations

ANBS: Australian National Breastfeeding Strategy
BFHI: Baby Friendly Health Initiative
B-L model: The Burke and Litwin model (1992)
CFHN: Child and Family Health Nurse
HMBs: Human Milk Banks
IBCLC: International Board-Certified Lactation Consultant
MAIF Agreement: Marketing in Australia of Infant Formula Agreement
NICU: Neonatal Intensive Care Unit
RM: Registered Midwife
RN: Registered Nurse
UNICEF: United Nations International Children’s Emergency Fund
WHO: World Health Organization
WHO Code: The International Code of Marketing of Breast-milk Substitutes
